# Organ-specific off-target effects of Pim/ZIP kinase inhibitors suggest lack of contractile Pim kinase activity in prostate, bladder, and vascular smooth muscle

**DOI:** 10.1007/s00210-023-02664-6

**Published:** 2023-09-01

**Authors:** Sheng Hu, Moritz Trieb, Ru Huang, Alexander Tamalunas, Patrick Keller, Melanie Götz, Raphaela Waidelich, Christian G. Stief, Martin Hennenberg

**Affiliations:** 1 Department of Urology, University Hospital Munich, LMU Munich, Munich, Germany; 2 Urologische Klinik Und Poliklinik, Marchioninistr. 15, 81377 München, Germany

**Keywords:** ZIPK, Pim kinase, Benign prostatic hyperplasia (BPH), Lower urinary tract symptoms (LUTS), Prostate smooth muscle, Bladder smooth muscle, Vasocontraction

## Abstract

Smooth muscle contraction by Pim kinases and ZIPK has been suggested, but evidence for lower urinary tract organs or using Pim-selective inhibitor concentrations is not yet available. Here, we assessed effects of the Pim inhibitors AZD1208 and TCS PIM-1 and the dual ZIPK/Pim inhibitor HS38 on contractions of human prostate and bladder tissues and of porcine interlobar arteries. Human tissues were obtained from radical prostatectomy and radical cystectomy and renal interlobar arteries from pigs. Contractions were studied in an organ bath. Noradrenaline-, phenylephrine- and methoxamine-induced contractions were reduced (up to > 50%) with 500-nM AZD1208 in prostate tissues and to lesser degree and not consistently with all agonists in interlobar arteries. A total of 100-nM AZD1208 or 500-nM TCS PIM-1 did not affect agonist-induced contractions in prostate tissues. Decreases in agonist-induced contractions with 3-µM HS38 in prostate tissues and interlobar arteries were of small extent and did not occur with each agonist. Carbachol-induced contractions in detrusor tissues were unchanged with AZD1208 (500 nM) or HS38. Electric field stimulation–induced contractions were not affected with AZD1208 or HS38 in any tissue, but slightly reduced with 500-nM TCS PIM-1 in prostate tissues. Concentration-dependent effects of Pim inhibitors suggest lacking Pim-driven smooth muscle contraction in the prostate, bladder, and interlobar arteries but point to organ-specific functions of off-targets. Procontractile functions of ZIPK in the prostate and interlobar arteries may be limited and are lacking in the detrusor.

**Supplementary Information:**

The online version contains supplementary material available at 10.1007/s00210-023-02664-6.

## Introduction

Smooth muscle contraction in the lower urinary tract and cardiovascular system holds a pivotal position in pathophysiology and medical treatment of widespread diseases, including lower urinary tract symptoms (LUTS), arterial hypertension, and diabetic nephropathy. Worldwide, numbers of LUTS patients have been extrapolated up to 2.2 billion for 2018, while annual deaths have been recently estimated up to 10.4 million for elevated systolic blood pressure and to 18.8 million for any cardiovascular disease (Irwin et al. 2011; Roth et al. 2020; Zhou et al 2021). First-line options for medical LUTS treatment include α_1_-adrenoceptor antagonists for voiding symptoms attributed to benign prostatic hyperplasia (BPH) and muscarinic receptor antagonists for storage symptoms in overactive bladder (OAB), believed to improve symptoms by inhibition of α_1_-adrenergic smooth muscle contraction in the prostate or of cholinergic contractions in the bladder (Gravas et al. 2023; Michel et al. 2023). Gold standard options for treatment of cardiovascular diseases are based on drugs inducing vasorelaxation (Brouwers et al. 2021), whereas hypotensive side effects may limit medical LUTS treatment, in parallel to insufficient symptom improvements (Gravas et al. 2023). In the face of high burdens to patients and health care systems, and considering limitations of available drugs and overlapping drug actions in different organs, understanding the principles of smooth muscle contraction in the lower urinary tract and cardiovascular system is of obvious relevance.

Previous findings from vascular and gastrointestinal smooth muscle repeatedly suggested a role of ZIP kinase (ZIPK, syn. death-associated protein kinase 3, DAPK3) for promotion of smooth muscle contraction (Haystead 2005; Ihara and MacDonald 2007; Deng et al. 2019; Ihara et al. 2009; Komatsu and Ikebe 2014). However, evidence supporting or disproving an analog function in the prostate or bladder has not yet been reported. Inhibition of smooth muscle contraction by HS38, observed in isolated vascular and ileal rodent tissues, has been initially explained by inhibition of ZIPK and DAPK1 (Carlson et al. 2013; MacDonald et al. 2016); before inhibition of Pim kinases, specifically Pim-1 and -3 by HS38, and an involvement in anticontractile effects has been supposed as well (Carlson et al. 2018). Recent attempts aimed to develop inhibitors with higher selectivity for Pim kinases as for DAPKs. However, available data describing effects of presumed Pim kinase inhibitors on smooth muscle contraction are currently limited to one study in vascular smooth muscle, using a concentration not being selective for Pim kinases, but also inhibiting all three DAPKs (Carlson et al. 2018). Thus, even though a procontractile role of Pim kinases has been proposed and Pim kinases were considered as a promising target for antihypertensive drugs (Carlson et al. 2018), conclusive data supporting Pim kinase functions in smooth muscle contraction are in fact still pending.

Considering the presumed but uncertain function of Pim kinases in vasocontraction, and that analog roles of ZIPK or Pim kinases have not been considered in the lower urinary tract smooth muscle contraction, we were interested to examine effects of inhibitors targeting ZIPK and Pim kinases on prostate and bladder smooth muscle contraction, using concentrations selective for ZIPKs and Pim kinases. Here, we examined effects of AZD1208, TCS PIM-1 1, and HS38 on agonist-induced and neurogenic contractions of human prostate and detrusor tissues and of porcine renal interlobar arteries.

## Materials and methods

### Human prostate tissues

Human prostate tissues were obtained from radical prostatectomy for prostate cancer. Prostates from patients with previous ablative surgery for BPH were excluded. This study was carried out in accordance with the Declaration of Helsinki of the World Medical Association and has been approved by the ethics committee of Ludwig Maximilian University, Munich, Germany. Informed consent was obtained from all patients. All samples and data were collected and analyzed anonymously. Approximately 30–60 min after removal of prostates from patients, macroscopical inspection and sampling were performed by a pathologist. For storage and transport, organs and samples were stored in Custodiol^®^ solution (Köhler, Bensheim, Germany). For macroscopic inspection and sampling, the prostate was opened by a single longitudinal cut from the capsule to the urethra, and both intersections were checked macroscopically for any obvious tumor infiltration. Subsequently, tissues were taken from the transitional, periurethral zone. Prostates showing tumors in the periurethral zone during macroscopic inspection were excluded. In fact, this was rare (< 1% of prostates), as most tumors in the prostate arise in the peripheral zone (Pradidarcheep et al. 2011; Shaikhibrahim et al. 2012). Organ bath experiments were started within 3 h after sampling.

### Human detrusor tissues

Human tissues from the lateral bladder wall were obtained from radical cystectomy for bladder cancer. This study was carried out in accordance with the Declaration of Helsinki of the World Medical Association and has been approved by the ethics committee of Ludwig Maximilian University, Munich, Germany. Informed consent was obtained from all patients. All samples and data were collected and analyzed anonymously. Accordingly, no patients’ data were collected, stored, or analyzed in the context of this study. Macroscopical inspection and sampling by a pathologist were performed within approximately 30–60 min after removal of bladders from patients. For macroscopic inspection and sampling, bladders were opened by a longitudinal cut from the bladder outlet to the bladder dome. Subsequently, the bladder wall and intravesical surface were macroscopically checked for tumor infiltration. Provided that tumor burden in the bladder wall allowed sampling, tissues were taken from the inner lateral bladder wall, and urothelial layers were resected from samples. Experiments were started not later than 60 min after sampling. For storage and transport, organs and tissues were placed in Custodiol^®^ solution.

### Porcine interlobar arteries

Porcine kidneys were obtained from a local slaughterhouse, where pigs were sacrificed during night for meat production. Organs were picked up by a butcher still during night, stored at 4 °C and transported in the early morning from the butcher’s shop (Metzgerei Brehm, Planegg, Germany) to the nearby investigators’ laboratory. Segments of interlobar arteries were prepared from organs immediately after arrival in the laboratory. Connective and adipose tissue surrounding the vessels was removed, and segments were cut into rings (3–4 mm in length), which were stored in Custodiol^®^ solution (Köhler, Bensheim, Germany) at 4 °C until being used for experiments. Experiments were started within 3 h following vessel preparation.

### Organ bath experiments

Prostate or detrusor strips (6 × 3 × 3 mm) and vessel rings with a length of 2–3 mm were mounted in organ baths, with four chambers in each device (model 720 M, Danish Myo Technology, Aarhus, Denmark). Each chamber contained 10-ml Krebs–Henseleit solution (37 °C, pH 7.4), which was continuously aerated with carbogen (95% O_2_ and 5% CO_2_) during the whole experiment. Arterial segments were stretched to 10 mN and prostate and bladder tissues to 4.9 mN. As pretensions spontaneously decline in the early phase after mounting, tensions were readjusted in the next 45 min, resulting in the stable, intended pretension. For later reference of agonist-induced and neurogenic contractions to high molar KCl-induced contractions, tissues were subsequently contracted by addition of a 2 M KCl solution to organ bath chambers, resulting in a final potassium concentration of 80 mM. As soon as a plateau or maximum contraction was obviously obtained, the solution in the chambers was replaced by new Krebs–Henseleit solution, three times within 30 min. After a new, stable baseline was obtained, inhibitors or solvent for controls were added. Frequency response curves for electric field stimulation (EFS) or cumulative concentration response curves for agonists were constructed after further 30 min.

Each independent experiment was performed using tissue from the same organ and included an inhibitor and a control group. Only one concentration response or frequency response curve was recorded with each sample. Wherever possible, double determinations were performed. For double determinations, two organ bath channels were examined with inhibitor and the two others in the same device with solvent. From a total of 181 experiments, double determinations in both groups were possible in 146 experiments. In the remaining experiments, the amount of sampled tissues did not allow filling of two channels for both groups, or single samples did not contract with KCl, so that single determinations were performed in one group, or rarely in both groups. However, each experiment contained at least one sample for both groups, resulting in paired samples. Allocations of channels to control and inhibitor groups were changed between experiments.

Agonist- and EFS-induced contractions are expressed as percentage of 80-mM KCl-induced contractions, as this may correct varying phenotypes and degrees of BPH or OAB or any individual variations and heterogeneities. *E*_max_ values, *EC*_50_ values for agonists, and frequencies (*f*) inducing 50% of the maximum EFS-induced contraction (*Ef*_50_) were calculated separately for each single experiment by curve fitting as previously described (Huang et al. 2022), using GraphPad Prism 6 (GraphPad Software Inc., San Diego, CA, USA). Error messages, sent by the program if curve fitting is not possible or if results from curve fitting are suspected as “ambiguous” or non-plausible, occurred in experiments applying carbachol to detrusor tissues, but in no other series. Consequently, high carbachol concentrations were excluded for curve fitting in the control group for AZD1208 in one experiment with carbachol, in the control group for HS38 in another experiment with carbachol, and in two HS38 groups again in two carbachol experiments (see supplementary information files). In addition, values from curve fitting were checked manually for plausibility, as recommended in the “GraphPad Curve Fitting Guide” (GraphPad Software, Inc., San Diego, CA, USA). Several values were marked as “ambiguous” in carbachol experiments, despite exclusion of downhill parts in curve fitting as described above, but were included in all analyses after manual review.

### Materials, drugs, and nomenclature

HS38, AZD1208, and TCS PIM-1 1 are small molecule kinase inhibitors, with specificities, targets, and off-targets summarized below (see the “Discussion” section for details). In brief, kinases inhibited by AZD1208 with *K*_d_ values below 10 µM include all three Pim isoforms, DAPK1, and at least five other kinases (Keeton et al. 2014). TCS PIM-1 1 inhibits Pim-1 with an *IC*_50_ of 50 nM, while further data regarding its specificity are obviously not available (Cheney et al. 2007). Data reported by the provider and suggesting inhibition of Pim-2 and MEK1/2 by TCS PIM-1 1, each with *IC*_50_ values > 20 µM, could not be verified by own literature searches or on request by the provider and are not supported by the referenced publication (Cheney et al. 2007). HS38 may inhibit DAPK1 and -2, ZIPK, Pim-1 to -3, IRAK4, and DYRK2 using concentrations below 5 µM (Carlson et al. 2013; Carlson et al. 2018). Phenylephrine ((R)-3-[-1-hydroxy-2-(methylamino)ethyl]phenol) and methoxamine (α-(1-aminoethyl)-2,5-dimethoxybenzyl alcohol) are α_1_-selective adrenoceptor agonists. Aqueous stock solutions (10 mM) of noradrenaline, phenylephrine, and methoxamine were freshly prepared before each experiment. AZD1208, TCS PIM-1 1, and HS38 were obtained from Tocris (Bristol, UK), with HS38 meanwhile (i.e., during manuscript preparation) being discontinued. Noradrenaline, phenylephrine, and methoxamine were obtained from Sigma-Aldrich (Munich, Germany). DMSO, KCl, and all other chemicals required for the preparation of Krebs–Henseleit solution were obtained from Carl Roth (Karlsruhe, Germany).

### Data and statistical analyses

Data in concentration and frequency response curves are means ± standard deviation (SD). *E*_max_, *EC*_50_, and *Ef*_50_ values are presented as single values (means from double determination, where this was possible), partly together with means from all independent experiments in scatter plots. Inhibitions at single agonist concentrations and single frequencies, calculated by normalization of values with inhibitor to the corresponding control in each single experiment and expressed as means with 95% confidence intervals (CIs), are additionally reported as percent inhibition with 95% CIs in a table. Calculation of 95% CIs and statistical analyses were performed using GraphPad Prism 6. Comparison of whole curves was performed by two-way analysis of variance (ANOVA), without multiple comparison, as previously described (Huang et al. 2022). Post hoc analyses for multiple comparisons at single agonist concentrations or frequencies were not performed, as this has been discouraged by the “GraphPad Statistics Guide” (GraphPad Software Inc., San Diego, CA, USA). *E*_max_, *EC*_50_, and *Ef*_50_ values were compared by a paired Student’s *t*-test. *p* values < 0.05 were considered significant. *p* values ≥ 0.05 have not been indicated. The present study and analyses show an exploratory design, as typical features of a strictly hypothesis-testing study were lacking, including a clear preset study plan, blinding, or biometric calculation of group sizes (Michel et al. 2020). Consequently, *p* values reported here need to be considered as descriptive, but not as hypothesis-testing (Michel et al. 2020). Interpretation and discussion of results was based on effect sizes and their possible relevance, instead of *p* values. Minimum group sizes were pre-planned as *n* = 5 for each series, to allow calculation of descriptive *p* values. Thus, series were discontinued after five independent experiments, if it was obvious that no effect could be expected or if *p* values were < 0.05 between both groups in frequency/concentration response curves. If these initial results were inconclusive, i.e., pointed to a possible drug effect but with *p* values > 0.05, series were continued and analyzed again. Specifically, increasing group sizes after five initial experiments was applied to experiments with TCS PIM-1 1 and EFS and to all experiments with interlobar arteries. This procedure was possible due to the explorative character and as long as it is reported in detail (Michel et al. 2020). Interim analyses were limited to frequency and concentration response curves and did not include *E*_max_, *EC*_50_, and *Ef*_50_ values, which were calculated only after completion of series. No data or experiments were excluded from analyses, apart from downhill parts in concentration response curves with carbachol during curve fitting.

## Results

### Effects of AZD1208 on contractions of human prostate tissues

**Fig. 1:**
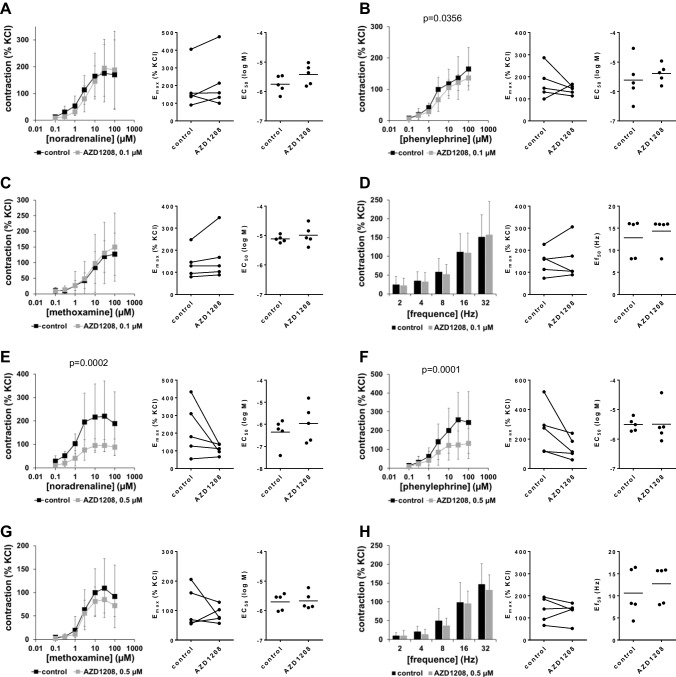
Effects of AZD1208 on adrenergic and EFS-induced contractions of human prostate tissues. Contractions of human prostate tissues were induced 30 min after addition of 100-nM AZD1208 **(A–D)**, 500-nM AZD1208 **(E–H)**, or of an equivalent amount of DMSO (controls) by noradrenaline **(A, E)**, phenylephrine **(B, F)**, methoxamine **(C, G)**, or EFS **(D, H)** in an organ bath. Shown are data from *n* = 5 independent experiments in each panel, where tissues from *n* = 5 patients were used for both groups of a subpanel, resulting in paired samples. Shown are means ± SD from all experiments in concentration response curves, together with *p* values from two-way ANOVA for whole groups and all single *E*_max_, *EC*_50_, and *Ef*_50_ values from all experiments (calculated by curve fitting). Corresponding *E*_max_ values, i.e., control and inhibitor values obtained from the same tissue, are connected by lines, with overlapping points indicating similar values, obtained using different tissues

Contractions induced by noradrenaline, phenylephrine, methoxamine, or EFS in human prostate tissues were not changed by 0.1-µM AZD1208, neither in concentration or frequency response curves nor the *E*_max_, *EC*_50_, or *Ef*_50_ values (Fig. 1A–D). Using 0.5-µM AZD1208 contractions by all three α_1_-adrenergic agonists were reduced (Fig. 1E–G). Decreases of α_1_-adrenergic contractions were largest with noradrenaline and phenylephrine, weak with methoxamine, and reflected by reduced *E*_max_ values (Fig. 1E–G, Table 1). *EC*_50_ values for α_1_-adrenergic agonists and EFS-induced contractions were unchanged with 0.5-µM AZD1208 (Fig. 1E–H).

**Table 1:** Agonist-induced contractions with AZD1208, HS38, and TCS PIM-1 1 (in indicated concentrations), expressed as percentage decreases from corresponding controls

			Agonist concentration				
			1 µM	3 µM	10 µM	30 µM	100 µM
AZD1208, 0.5 µM	Prostate	Noradrenaline	50% [− 3 to 103]	42% [− 17 to 101]	37% [− 10 to 83]	37% [− 8 to 82]	10% [− 100 to 119]
		Phenylephrine	12% [− 100 to 125]	14% [− 97 to 126]	35% [− 21 to 90]	49% [11 to 86]	40% [8 to 74]
		Methoxamine	86% [11 to 162]	− 22% [− 173 to 129]	6% [− 58 to 71]	3% [− 67 to 74]	− 12% [− 98 to 73]
	Interlobar artery	Noradrenaline	− 19% [− 59 to 22]	− 11% [− 44 to 22]	− 2% [− 37 to 33]	0% [− 37 to 37]	− 1% [− 40 to 38]
		Phenylephrine	− 30% [− 126 to 65]	13% [− 10 to 35]	19% [0 to 39]	17% [− 9 to 43]	21% [1 to 41]
		Methoxamine	− 1% [− 29 to 27]	− 3% [− 43 to 36]	− 4% [− 47 to 39]	− 9% [− 54 to 36]	− 9% [− 56 to 39]
HS38, 3 µM	Prostate	Noradrenaline	− 21% [− 195 to 154]	15% [− 62 to 91]	5% [− 34 to 43]	19% [− 5 to 43]	27% [0 to 54]
		Phenylephrine	40% [− 62 to 143]	18% [− 38 to 74]	17% [− 13 to 47]	2% [− 20 to 24]	− 3% [− 55 to 48]
		Methoxamine	0% [− 131 to 131]	− 22% [− 227 to 184]	17% [− 40 to 74]	1% [− 54 to 55]	20% [1 to 38]
	Interlobar artery	Noradrenaline	− 10% [− 44 to 25]	− 7% [− 35 to 21]	− 7% [− 32 to 18]	− 4% [− 28 to 19]	− 5% [− 28 to 18]
		Phenylephrine	19% [− 17 to 55]	38% [− 18 to 58]	30% [9 to 51]	28% [7 to 48]	26% [2 to 49]
		Methoxamine	− 20% [− 129 to 89]	11% [− 29 to 51]	6% [− 21 to 33]	6% [− 20 to 31]	6% [− 18 to 31]
TCS PIM-1 1, 0.5 µM	Prostate	Noradrenaline	27% [− 37 to 91]	28% [− 13 to 70]	13% [− 22 to 48]	4% [− 29 to 37]	14% [3 to 24]
		Phenylephrine	− 51% [− 207 to 104]	25% [− 39 to 90]	15% [− 24 to 54]	− 3% [− 65 to 58]	− 14% [− 57 to 29]
		Methoxamine	− 277% [− 1214 to 661]	− 143% [− 655 to 370]	12% [− 59 to 84]	2% [− 62 to 66]	− 8% [− 97 to 82]

For each single experiment, contractions with inhibitor were calculated as percent of the corresponding control in the same experiment and subtracted from the control (100-(contraction with inhibitor)/(contraction control)*100). Data are means from all single experiments together with 95% CI

### Effects of HS38 on contractions of human prostate tissues

**Fig. 2:**
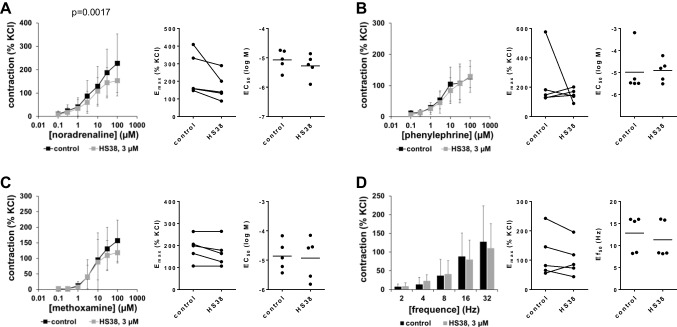
Effects of HS38 on adrenergic and EFS-induced contractions of human prostate tissues. Contractions of human prostate tissues were induced by noradrenaline **(A)**, phenylephrine **(B)**, methoxamine **(C)**, or EFS **(D)**, in an organ bath, 30 min after addition of 3-µM HS38 or of an equivalent amount of DMSO (controls). Shown are data from *n* = 5 independent experiments in each panel, where tissues from *n* = 5 patients were used for both groups of a subpanel, resulting in paired samples. Shown are means ± SD from all experiments in concentration response curves, together with *p* value from two-way ANOVA for whole groups, and all single *E*_max_, *EC*_50_, and *Ef*_50_ values from all experiments (calculated by curve fitting). Corresponding *E*_max_ values, i.e., control and inhibitor values obtained from the same tissue, are connected by lines, with overlapping points indicating similar values, obtained using different tissues

Using 3-µM HS38, contractions induced by noradrenaline, phenylephrine, and methoxamine in human prostate tissues were partly but not consistently reduced (Fig. 2A–C, Table 1). Reduced contractions were observed at the highest applied concentrations of noradrenaline and methoxamine, to slight extent but being reflected by reduced *E*_max_ values for noradrenaline (Fig. 2A, Table 1), and to even smaller or possibly neglectable extent for methoxamine (Fig. 2C, Table 1). EFS-induced contractions were not changed by 3-µM HS38 (Fig. 2D).

### Effects of TCS PIM-1 1 on contractions of human prostate tissues

**Fig. 3:**
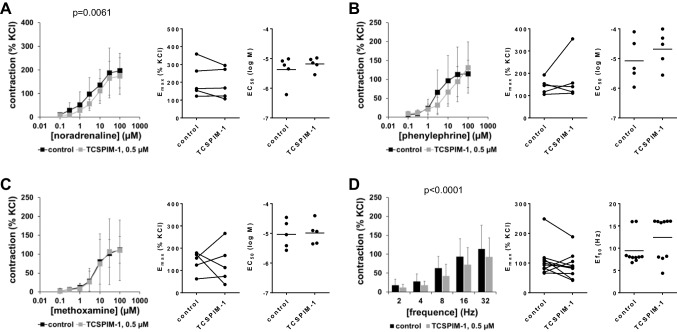
Effects of TCS PIM-1 1 on adrenergic and EFS-induced contractions of human prostate tissues. Contractions of human prostate tissues were induced by noradrenaline **(A)**, phenylephrine **(B)**, methoxamine **(C)**, or EFS **(D)** in an organ bath, 30 min after addition of 500-nM TCS PIM-1 1 or of an equivalent amount of DMSO (controls). Shown are data from *n* = 5 independent experiments in (**A**)–(**C**) and *n* = 10 independent experiments in (**D**), where tissues from *n* = 5 or *n* = 10 patients were used for both groups of a subpanel, resulting in paired samples. Shown are means ± SD from all experiments in concentration response curves, together with *p* value from two-way ANOVA for whole groups, and all single *E*_max_, *EC*_50_, and *Ef*_50_ values from all experiments (calculated by curve fitting). Corresponding *E*_max_ values, i.e., control and inhibitor values obtained from the same tissue, are connected by lines, with overlapping points indicating similar values, obtained using different tissues

Using 0.5-µM TCS PIM-1 1, contractions by noradrenaline or phenylephrine were reduced marginally, if at all in human prostate tissues (Fig. 3A, B, Table 1). Decreases were not paralleled by reduced *E*_max_ values (Fig. 3A, B). Contractions induced by methoxamine were unchanged with TCS PIM-1 1 (Fig. 3C, Table 1). EFS-induced contractions were reduced with 0.5-µM TCS PIM-1 1 (Fig. 3D, Table 1).

### Effects of AZD1208 on contractions of porcine interlobar renal arteries

**Fig. 4:**
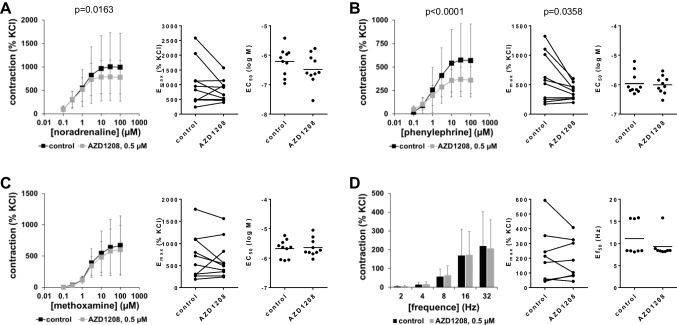
Effects of AZD1208 on adrenergic and EFS-induced contractions of porcine renal interlobar arteries. Contractions were induced by noradrenaline **(A)**, phenylephrine **(B)**, methoxamine **(C),** or EFS **(D)** in an organ bath, 30 min after addition of 500-nM AZD1208 or of an equivalent amount of DMSO (controls). Shown are data from *n* = 10 independent experiments in (**A**), (**B**), and (**C**) and *n* = 8 independent experiments in (**D**), where tissues from *n* = 10 or *n* = 8 animals were used for both groups of a subpanel, resulting in paired samples. Shown are means ± SD from all experiments in frequency and concentration response curves, together with *p* values from two-way ANOVA for whole groups, and all single *E*_max_, *EC*_50_, and *Ef*_50_ values from all experiments (calculated by curve fitting) together with *p* value from Student’s *t*-test in scatter plots. Corresponding *E*_max_ values, i.e., control and inhibitor values obtained from the same tissue, are connected by lines, with overlapping points indicating similar values, obtained using different tissues

Contractions of porcine interlobar arteries by α_1_-adrenergic agonists were partly reduced with 0.5-µM AZD1208, even though not consistently across all three agonists (Fig. 4A–C, Table 1). Contractions by noradrenaline were slightly reduced with AZD1208 (Fig. 4A, Table 1). An obvious, larger decrease of phenylephrine-induced contractions seen in concentration response curves was paralleled by reduced *E*_max_ values for phenylephrine (Fig. 4B, Table 1). Discrete from decreases in noradrenaline- and phenylephrine-induced contractions, contractions induced by methoxamine were unchanged with AZD1208 (Fig. 4C, Table 1). EFS-induced contractions of porcine interlobar arteries were not affected by 0.5-µM AZD1208 (Fig. 4D).

### Effects of HS38 on contractions of porcine interlobar renal arteries

**Fig. 5:**
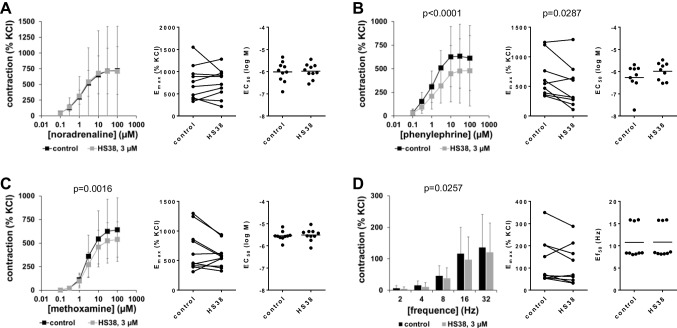
Effects of HS38 on adrenergic and EFS-induced contractions of porcine renal interlobar arteries. Contractions were induced by noradrenaline **(A)**, phenylephrine **(B)**, methoxamine **(C)**, or EFS **(D)** in an organ bath, 30 min after addition of 3 µM HS38 or of an equivalent amount of DMSO (controls). Shown are data from *n* = 10 independent experiments in (A) and (C) and *n* = 9 independent experiments in (B) and (D), where tissues from *n* = 10 or *n* = 9 animals were used for both groups of a subpanel, resulting in paired samples. Shown are means ± SD from all experiments in frequency and concentration response curves, together with *p* values from two-way ANOVA for whole groups, and all single *E*_max_, *EC*_50_, and *Ef*_50_ values from all experiments (calculated by curve fitting) together with *p* value from Student’s *t*-test in scatter plots. Corresponding *E*_max_ values, i.e., control and inhibitor values obtained from the same tissue, are connected by lines, with overlapping points indicating similar values, obtained using different tissues

Contractions of porcine interlobar arteries by α_1_-adrenergic agonists were partly reduced with 3-µM HS38, i.e., at most to limited degree and not consistently with all three agonists (Fig. 5A–C, Table 1). Noradrenaline-induced contractions were not changed by 3-µM HS38 (Fig. 5A, Table 1). A partial, but obvious, decrease of phenylephrine-induced contractions seen in concentration response curves was paralleled by reduced *E*_max_ values for phenylephrine (Fig. 5B, Table 1). Contractions by methoxamine were slightly reduced with HS38 (Fig. 5C, Table 1). EFS-induced contractions were not affected by 3-µM HS38 (Fig. 5D).

### Effects of HS38 and AZD1208 on contractions of human detrusor tissues

**Fig. 6:**
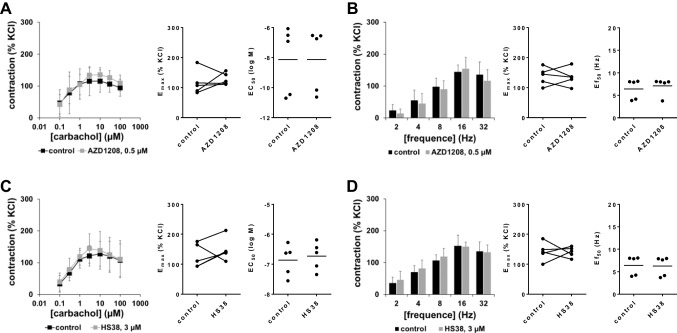
Effects of AZD1208 and HS38 on carbachol-induced and EFS-induced contractions of human detrusor tissues. Contractions of human detrusor tissues were induced 30 min after addition of 500-nM AZD1208 **(A, B)**, 3-µM HS38 **(C, D)**, or of an equivalent amount of DMSO (controls) by carbachol **(A, C)** or EFS **(B, D)** in an organ bath. Shown are data from *n* = 5 independent experiments in each panel, where tissues from *n* = 5 patients were used for both groups of a subpanel, resulting in paired samples. Shown are means ± SD from all experiments in concentration response curves, and all single *E*_max_, *EC*_50_, and *Ef*_50_ values from all experiments (calculated by curve fitting). Corresponding *E*_max_ values, i.e., control and inhibitor values obtained from the same tissue, are connected by lines, with overlapping points indicating similar values, obtained using different tissues

Contractions induced by EFS or by carbachol in human detrusor tissues were neither affected by 0.5-µM AZD1208 (Fig. 6A, B) nor by 3-µM HS38 (Fig. 6C, D).

## Discussion

Our findings allow several conclusions regarding recently emerging Pim kinase functions and previously presumed ZIPK functions in smooth muscle contraction. Most evident was an inhibition of contractions induced by α_1_-adrenergic agonists in human prostate tissues by 500-nM AZD1208, which was consistently observed with all three examined agonists. As this did not occur with 100 nM, where inhibition of all three Pim kinase isoforms may be expected (Table 2), anticontractile effects of 300 nM were probably rather imparted by inhibition of kinases belonging to the off-target spectrum, rather than Pim kinases. In interlobar arteries, decreases in α_1_-adrenergic contractions by 500-nM AZD1208 were less obvious compared to prostate tissues, but not completely lacking. A recently proposed function of Pim kinases in promoting vascular smooth muscle contraction may therefore not necessarily be generalized to all vessel types or to all species. Decreases of α_1_-adrenergic contractions by 3-µM HS38 were again of limited extent and not observed with all three agonists, although effects were not fully absent in interlobar arteries and prostate tissues, suggesting a low or even lacking relevance of ZIPK functions in smooth muscle contraction of these tissues (Table 2). The relevance of Pim kinase and ZIPK, but also of off-targets of AZD1208 for smooth muscle contraction, may be organ-specific, as no effects were observed with AZD1208 or HS38 in human detrusor tissues.

**Table 2:** Targets and off-targets of AZD1208 and HS38. Possible (off-)targets involved in anticontractile effects observed with AZD1208 and HS38 in this study, suggested by combination and alignment of previous data for affinities and IC_50_ values, with applied concentrations and findings in our experiments. See main text for references reporting available affinities and inhibition data from pharmacological profiling of both compounds

	AZD1208		HS38			
	*K*_d_^1^	*IC*_50_^2^	*K*_d_	*IC*_50_^3^	Inhibition (10 µM) ^3^	*K*_i_
Pim-1	0.2 nM	2.6 nM	1.8 µM	1.7 µM	77% ^3^, ca. 60% ^4^	2.94 µM
Pim-2	0.88 nM	150–164 nM	6.5 µM	18 µM	36% ^3^, ca. 10% ^4^	> 100 µM
Pim-3	0.76 nM	9–17 nM	810 nM	200 nM	76% ^3^, ca. 85% ^4^	208 nM
DAPK1	420 nM		300 nM	200 nM	89% ^3^	
DAPK2	Probably > > 10 µM		79 nM			
ZIPK/DAPK3	Probably > > 10 µM		280 nM		ca. 85% ^4^	260 nM
MLCK	Probably > > 10 µM			19 µM	29% ^3^	
ROCK	Probably > > 10 µM			200 µM	9% ^3^	
Off-targets	CDK7 38 nM, MAPK15 53 nM, CAMK4 360 nM, HIPK3 480 nM, STK17B 930 nM, seven further kinases > 10 µM			IRAK4 1.4 µM, DYRK2 1.8 µM	IRAK4 80%, DYRK2 79% 3	
Prostate tissues	α_1_-adrenergic contractions inhibited by 500 nM, but not by 100 nM		α_1_-adrenergic contractions only slightly and partly inhibited by 3 µM			
Interlobar arteries	α_1_-adrenergic contractions partly inhibited by 500 nM		α_1_-adrenergic contractions partly inhibited by 3 µM			
Putative (off-)targets involved in anticontractile effects ^5^	DAPK1, CAMK4, HIPK3 (K_d_/IC_50_ > > 100 nM, < 900 nM). Pim kinases: no, because no inhibition with 100 nM		ZIPK, IRAK4, DYRK2 (inhibition with 3 µM expected). Pim-1, -3: no, because no inhibition by 100 nM AZD1208. DAPK1: probably no, because no inhibition in prostate, but possible target for AZD1208 in prostate, while targets of AZD1208 and HS38 must be unshared between prostate and interlobar artery			

^1^Determined in follow-up, after 13 off-target kinases were identified (inhibited > 50% by unknown concentration of AZD1208) by screening of 442 kinases

^2^In biochemical assays with 5-mM ATP (= high end of physiologic ATP concentrations in human cells)

^3^Biochemical assays using 124 different, purified kinases, not including ZIPK

^4^Biochemical assay, analog to (3)

^5^By fusion and intersection of organ bath data in this study with data from pharmacological profiling

AZD1208 has been developed as a Pim-selective inhibitor (Table 2), showing *IC*_50_ values of 2.6 nM for Pim-1, 150–164 nM for Pim-2, and 9–17 nM for Pim-3, in biochemical assays with 5-mM ATP, representing the high end of physiologic ATP concentrations in human cells (Keeton et al. 2014; Dakin et al. 2012). Pim-1 to -3 were the kinases being inhibited strongest by AZD1208 in a screening assay including 442 kinases, while activities of further 13 kinases were inhibited by 50% or more (Keeton et al. 2014). However, no information is available about degrees of inhibitions or about the applied concentration of AZD1208 (Keeton et al. 2014). *K*_d_ values of AZD1208 for these 16 kinases were determined in follow-up experiments, where they amounted to 0.2 nM for Pim-1, 0.76 nM for Pim-3, 0.88 nM for Pim-2, 38 nM for CDK7, 53 nM for MAPK15, 360 nM for CAMK4, 420 nM for DAPK1, 480 nM for HIPK3, 930 nM for STK17B, and > 10 µM for the seven remaining kinases (Keeton et al. 2014).

Considering that *K*_d_ and *IC*_50_ values for Pim kinases mostly ranged clearly below 100 nM, while 100 nM did not affect contractions in prostate tissues, the anticontractile effects of 500 nM in prostate tissues and interlobar arteries did most probably not involve inhibition of Pim kinases. Consequently, DAPK1, CAMK4, and HIPK3 are possible candidates accounting for anticontractile effects observed with 500 nM, as *K*_d_ values of AZD1208 for these off-targets range higher than 100 nM (Table 2). An involvement of other known off-targets may be excluded, as *K*_d_ values either range below 100 nM or higher than 900 nM, which does not match the concentration-inhibition profile in our organ bath experiments. Together, our findings do not support a role of Pim kinases in promotion or regulation of smooth muscle contraction in the human prostate, human detrusor, or in porcine interlobar arteries.

A lacking role of Pim-1 in our examined tissues was confirmed using 500-nM TCS PIM-1 1. TCS PIM-1 1 inhibits Pim-1 with an *IC*_50_ of 50 nM and ATP competitively in biochemical assays (Cheney et al. 2007). Further data regarding other kinases and off-targets are obviously not available. Inhibition of Pim-2 and MEK by TCS PIM-1 1 with *IC*_50_ values > 20 µM, suggested by information on the supplier’s homepage, could not be verified, neither on request nor by literature research. Reduced EFS-induced contractions of prostate tissues, observed with TCS PIM-1 1, were not shared with AZD1208 or HS38, suggesting this was not imparted by Pim kinase inhibition. Rather, the discrete effect on EFS-induced contractions points to off-targets of TCS PIM-1 1, which are not shared by AZD1208 or HS38. A recently proposed role of Pim kinases in vasocontraction can obviously not be generalized to all vessel types or to all species. Pim kinase-mediated vasocontraction has been suggested by observations that a dual Pim-1/ZIPK inhibitor reduces blood pressure in hypertensive mice and impairs contractions in isolated rat caudal arteries, with higher efficacy as a ZIPK inhibitor without inhibitory activity on Pim-1 (Carlson et al. 2018).

HS38 has been introduced as an ATP-competitive small molecule inhibitor preferentially targeting DAPK1 and ZIPK (Carlson et al. 2013). In competition binding assays, HS38 bound with *K*_d_ values of 300 nM to DAPK1, 79 nM to DAPK2, 280 nM to ZIPK, 1.8 µM to Pim-1, 6.5 µM to Pim-2, and 810 nM to Pim-3 (Carlson et al. 2013). In biochemical assays using 124 different, purified kinases, but interestingly not including ZIPK, *IC*_50_ values for HS38 amounted to 200 nM for DAPK1 and Pim-3; 1.4–1.8 µM for IRAK4, Pim-1, and DYRK2; 18–19 µM for Pim-2 and MLCK; and > 200 µM for ROCK2 (Carlson et al. 2013). Using 10 µM, activities of DAPK1, Pim-3, IRAK4, Pim-1, and DYRK2 were inhibited by more than 75%, while 28 further kinases were inhibited between 25 and 80% (MLCK by 29%, ROCK2 by 9%) and ZIPK was not included to these assays (Carlson et al. 2013). Data including ZIPK have been reported in a follow-up study by the same group, reporting *K*_i_ values of HS38 of 260 nM for ZIPK, 208 nM for Pim-3, 2.94 µM for Pim-1, and > 100 µM for Pim-2 and inhibition of ZIPK and Pim-3 around 85%, of Pim-1 around 60%, and of Pim-2 around 10% by 10-µM HS38 (Carlson et al. 2018).

Considering these previously reported values and that we applied HS38 using a concentration of 3 µM, possible kinases involved in decreases of adrenergic contractions seen in our study may include ZIPK, DAPK1, Pim-1 and -3, IRAK4, DYRK2, and any other kinase not included in previous kinase assays. From these candidates, Pim-1 and -3 may be excluded as we did not observe inhibitory effects in experiments with 100-nM AZD1208 or with TCS PIM-1. DAPK1 is a shared off-target shared of HS38 and AZD1208, presumably inhibited by 500 nM, but not 100-nM AZD1208. As the effects of HS38 and AZD1208 were not fully consistent in prostate tissues, the inhibitory effects seen with 3-µM HS38 were possibly imparted by inhibition of ZIPK, IRAK4, or DYRK2, rather than DAPK1. Obviously and with high certainty, an involvement of these kinases, including ZIPK in human bladder smooth muscle contraction, can be excluded, as HS38 did not affect contractions of detrusor tissues. As α_1_-adrenergic contractions were reduced rather slightly by HS38, and not across all three examined agonists, the relevance of any ZIPK-driven contraction may be limited in human prostate smooth muscle and in porcine interlobar arteries. Nevertheless, our findings with renal interlobar vessels are in line with previous studies reporting inhibition of smooth muscle contraction by HS38, but using higher concentrations. Inhibitory effects have been reported using 10 µM in phenylephrine-induced contraction of mouse aortic tissue, 50 µM in carbachol- and calcium-induced contractions of rabbit ileum, and 100 µM in calcium-induced contraction of rat caudal arteries (Carlson et al. 2013), using 100 µM in contractions induced by phenylephrine, U46619, KCl, angiotensin-II, and endothelin-1 in rat caudal arteries (MacDonald et al. 2016), with 1 µM upward in contractions induced by serotonin or calcium in cerebral arteries from rats (Turner et al. 2023), and with 10 µM in myogenic contractions of human cerebral arteries (Turner et al. 2023).

The concentrations of AZD1208 applied in our study are comparatively low. As a major limitation, our study does not contain data confirming Pim inhibition in our tissues. Previous studies using AZD1208 in vitro applied highly varying, but mostly micromolar, concentrations. Concentration-dependent effects in cell cultures have been examined with 10–40 µM (Park et al. 2018; Yan et al. 2022), 1–10 µM (Kreuz et al. 2015; Harada et al. 2015), but also from 1 nM to 3 µM (Trigg et al. 2019). Experiments using single concentrations, again in cell cultures, often used concentrations from 1 to 5 µM (Kreuz et al. 2015; Harada et al. 2015; Cen et al. 2014; Scarpa et al. 2021). In a study addressing mechanisms imparting resistance to ALK inhibitors in eight different lines of neuroblastoma cells, *EC*_50_ values for decreases in cell viability varied from 10 to 100 nM in five cell lines and ranged around 1 µM in three others (Trigg et al. 2019). Accordingly, cellular responses in other studies were similar with 0.1- and 1-µM AZD1208 (Keeton et al. 2014), or with 2.5–10 µM (Harada et al. 2015), while Pim kinase inhibition was already maximum with 1 µM, and not further increased using 5–10 µM in non-Hodgkin lymphoma cells (Kreuz et al. 2015). Consequently, we suppose that Pim kinase inhibition likely occurred with 100–500 nM in our experiments. Larger inhibition of smooth muscle contraction (compared to our experiments) may not be excluded using higher, e.g., micromolar concentrations, but would be of limited conclusiveness regarding specific involvement of Pim kinase, owing to possible off-target effects. TCS PIM-1 1 has been previously applied in possibly only one study, where effects occurred from 12.5 µM upward, but not with lower concentrations, so that off-target effects may not be excluded (Seifert 2021).

While the spectrum of kinases possibly involved in inhibitory effects could be narrowed down to few candidates for both inhibitors, precise identification of involved kinases was not possible within this study and was out of its scope. Involvements of MLCK or ROCK-2, both well known as being essential for smooth muscle contraction (Somlyo and Somlyo 2003), can yet be excluded as concentrations required for their off-target inhibition are much higher than concentrations applied in our experiments (Table 2). Based on our findings, we cannot explain why inhibitions by AZD1208 and HS38 were limited to adrenergic agonists but did not include EFS-induced contractions, which are believed to result from adrenergic neurotransmission. We assessed contractions by α_1_-adrenergic agonists in prostate and arteries, and of carbachol in detrusor tissues, as the corresponding receptors are principal mediators of smooth muscle contraction in these organs and/or important targets for medical treatment (Gravas et al. 2023; Hennenberg et al. 2008; Touyz et al. 2018). α_1_-Adrenoceptor antagonists, muscarinic antagonists, and a panel of antihypertensive drugs are believed to improve LUTS in BPH or OAB or to reduce blood pressure by inhibition of smooth muscle contraction in the prostate, bladder, or cardiovascular system (Michel et al. 2023; Brouwers et al. 2021). However, benefits from these drugs are overall insufficient, resulting in high numbers of surgery for BPH, high burden to patients and socioeconomic costs of LUTS, and high numbers of deaths due to cardiovascular diseases (Zhou et al 2021; Michel et al. 2023; Hennenberg et al. 2014; Magistro and Stief 2020). In parallel, relationships between LUTS, hypertension, diabetes, and its complications, including renal dysfunction, become increasingly evident (Muderrisoglu et al. 2023). Even though compounds as AZD1208 are no candidates for clinical studies in this context, considering its side effects in oncological trials (Cortes et al. 2018), the need for ongoing attempts in understanding of shared and unshared molecular mechanisms of smooth muscle contraction in the lower urinary tract and cardiovascular system is obvious.

Parts of our data are characterized by high variability, which may be attributed to different maximum contraction levels, and to divergent effect sizes under identical inhibitor conditions, both varying between tissues from different patients or animals. Upregulation of α_1A_-adrenoceptors with age and BPH has been repeatedly reported at mRNA level and may in principle account for divergent contraction levels in prostate tissues but was not consistently supported by findings at protein level (Michel and Vrydag 2006). Expression levels of α_1_-adrenoceptors or muscarinic receptors in our tissues may be subject of individual variation, which is high and includes a panel of parameters, at least in human tissues. The age of patients undergoing radical prostatectomy at our department averages out at 66 ± 7 years (Grabbert et al. 2018), where prevalences range between 60 and 70% for histological BPH and between 25 and 40% for LUTS (Lepor 2004) and where LUTS treatment with α_1_-blockers may be common. In fact, around 80% of patients with prostate cancer show BPH (Alcaraz et al. 2009; Orsted and Bojesen 2013). Human tissues in our study were used anonymously, and without grouping or stratification, so that divergent degree of BPH and medical LUTS treatment may theoretically contribute to unknown extent to data variability. Clinical data related to LUTS are not systematically collected during hospitalization for radical prostatectomy at our department, so that these data are hardly available. However, we excluded patients with previous ablative surgery for BPH, as sampling from the periurethral zone is impossible in these prostates. Ablative surgery is mostly reserved to patients with severe LUTS, including patients with high risk of progression and for complications, with medication-refractory LUTS, or requesting treatment but refusing medication (Gravas et al. 2023). Consequently, the majority of patients included to collection of prostate tissues in our study may show BPH, and a high or substantial percentage may show mild to moderate LUTS, while probably only a small percentage may show severe LUTS and monotherapy with an α_1_-blocker was possibly the most common drug treatment for LUTS in this population. In principle, presence of an α_1_-blocker in our tissues (resulting from use of α_1_-blockers for LUTS treatment) could affect the tissue contractility and thus, the effect sizes resulting from inhibitor activities. However, this appears rather unlikely, as any compound in these tissues should have been washed out, first by Custodiol during transport and storage, and second during the equilibration period in the organ bath.

## Conclusions

Using concentrations selective and unselective for Pim kinases, our findings with AZD1208 point to a lacking role of Pim kinases in driving smooth muscle contraction in the prostate, bladder, and interlobar arteries but suggest organ-specific functions of off-targets. A lacking role of Pim kinases was confirmed using TCS PIM-1 1. Potential off-targets accounting for effects observed with a Pim kinase-unselective concentration of AZD1208 include DAPK1, CAMK4, and HIPK3, which have not yet been described in the context of smooth muscle contraction. Due to small and inconsistent effects of HS38, procontractile functions of ZIPK may be limited, if not lacking in the prostate and in interlobar arteries. In view of lacking effects in detrusor tissues, suggesting lacking ZIPK-driven contraction in bladder smooth muscle, together with previous findings, ZIPK functions in smooth muscle contraction may be organ-specific.

### Supplementary Information

Below is the link to the electronic supplementary material.

Supplementary file1 (XLSX 959 KB)

Supplementary file2 (ZIP 562 KB)

## Data Availability

Original and raw data containing all individual data points are available as supplemental information.
